# Extensive Behavioural Divergence following Colonisation of the Freshwater Environment in Threespine Sticklebacks

**DOI:** 10.1371/journal.pone.0098980

**Published:** 2014-06-10

**Authors:** Carole Di-Poi, Jennyfer Lacasse, Sean M. Rogers, Nadia Aubin-Horth

**Affiliations:** 1 Département de Biologie & Institut de Biologie Intégrative et des Systèmes (IBIS), Université Laval, Québec, Québec, Canada; 2 Department of Biological Sciences, University of Calgary, Calgary, Alberta, Canada; University of Basel, Switzerland

## Abstract

Colonisation of novel environments means facing new ecological challenges often resulting in the evolution of striking divergence in phenotypes. However, little is known about behavioural divergence following colonisation, despite the predicted importance of the role of behavioural phenotype-environment associations in adaptive divergence. We studied the threespine stickleback (*Gasterosteus aculeatus*), a model system for postglacial colonisation of freshwater habitats largely differing in ecological conditions from the ones faced by the descendants of the marine ancestor. We found that common-environment reared freshwater juveniles were less social, more active and more aggressive than their marine counterparts. This behavioural divergence could represent the result of natural selection that acted on individuals following freshwater colonisation, with predation as a key selection agent. Alternatively, the behavioural profile of freshwater juveniles could represent the characteristics of individuals that preferentially invaded freshwater after the glacial retreat, drawn from the standing variation present in the marine population.

## Introduction

The novel ecological challenges that individuals face when colonising new environments have been shown in many instances to result in adaptive trait divergence. Morphology has often been shown to diverge following colonisation as the result of natural selection [1]. Remarkable examples include the diversification of leg and tail lengths in anole lizards that use different microhabitats in trees and on land [2] and trophic morphology associated with different feeding ecology in African cichlid fishes [3]. Much less information is available on whether behaviours that could influence fitness in the new ecological context also evolve. This is true even for very well-known model systems of phenotypic divergence following colonisation of a novel environment. Yet, evolutionary changes in behaviour may be integral in initiating adaptive shifts [4]. It has been shown that behavioural divergence can be associated with morphological divergence in a new ecological context, whereby populations differ in the behavioural traits used to exploit different resources, such as in the association between beak shape and foraging behaviour in Darwin's finches [5]. The specific ecological constraints acting in the novel environment could directly affect the benefits associated with expressing a given behaviour. For example, when there are no substantial benefits associated with group life, such as in low-productivity freshwater habitats where predation pressure is weaker and intraspecific competition is the most relevant process affecting fitness, the advantageous strategy might be to adopt the solitary lifestyle and to increase foraging activity, boldness and aggressiveness towards conspecifics [6]–[8]. Boldness, exploratory behaviours and sociability (schooling) have been shown to diverge when individuals face new ecological conditions (poeciliid *Brachyraphis episcopi* [9]; *Poecilia reticulata* [10], [11]). Such behavioural divergence has been shown to enhance the probability of ecological speciation [12]. However, quantitative measurements of behavioural divergence between the ancestral population and the population in a new environment, potentially as the result of selection, remain scarce compared to other traits. Understanding the ecological context of behavioural divergence will help draw a complete picture of integrated phenotypes evolving in response to adaptive peak shifts [13].

To address this problem, we used a well-studied model system of colonisation of new environments, the threespine stickleback (*Gasterosteus aculeatus*). In the last 20,000 years, marine threespine sticklebacks have successfully colonised many freshwater systems with widely divergent ecological conditions in the Northern hemisphere [14]. A distinctive feature of this system is that the ancestral marine population is still extent, although it has also potentially diverged from the marine ancestor during this time period, allowing comparison with populations that have colonised new freshwater environments. Comparative studies between marine and freshwater populations have shown that colonisation of freshwater has resulted in rapid evolutionary changes in numerous traits in morphology (diminution of lateral armour and spine development, lower number of iridophores and presence of barred flanks [15], [16]), life history (younger and smaller at reproduction [17]), and physiology (lower levels of thyroid hormones, lower critical swimming speed [18], [19]). Furthermore, freshwater juveniles show higher territoriality [20] and a lower schooling tendency [21] than juvenile marine fish (representing the ancestral population). However, whether other ecologically relevant behaviours diverged following colonisation of the freshwater habitat by threespine sticklebacks has not been thoroughly studied.

We hypothesised that behaviour would have diverged between populations of sticklebacks that inhabit contrasting marine and freshwater environments [13], [22] since the costs and benefits of expressing a particular behaviour may vary according to ecological demands [21], [23]. We focused on genetically based differences in behaviour, rather than on phenotypic plasticity by studying common-environment reared juveniles originating from two populations of threespine sticklebacks representing the marine ancestor and the result of freshwater colonisation. We quantified boldness, aggressiveness, sociability, activity and exploratory behaviours, as they are relevant to fitness and ultimately to the ecology and evolution of populations [24]. Previous studies comparing two freshwater populations of sticklebacks differing in predation pressure have shown divergence in behaviour in sociability [25], activity [26], [27], aggressiveness [27], [28], and boldness [26], [28], [29]. Therefore, the potential behavioural divergence observed between marine and freshwater sticklebacks may be the result of natural selection, with predation as a key selection agent. Thus we predicted that sticklebacks from a freshwater lake population that experienced a presumed lower predation pressure based on their lower number of lateral armour plates would exhibit a lower sociability tendency, higher activity and exploratory behaviours, as well as a higher propensity to be aggressive towards a conspecific and to be bolder when foraging in a risky situation, compared to the individuals originating from the marine ancestor population that face a high-predation level environment.

## Methods

### Ethics statement

Wild animals were sampled under the Department of Fisheries and Ocean Permit #XR 315 2011 and the BC Ministry of Environment NA-SU-PE-10-63485 permit. The research adheres to the ASAB/ABS Guidelines for the Use of Animals in Research and was approved by the Comité de Protection des Animaux de l′Université Laval (permit #2010012-1).

### Sampling and rearing

Adult threespine sticklebacks from marine and freshwater populations were collected using minnow traps in British Columbia (Canada) in two breeding sites, which differ in fundamental ecological characteristics [13], [22]. Marine adult fish were collected from a small and shallow inlet off Malaspina Strait (Oyster Lagoon, 49°36′48′′N 124°01′47′′W) where salinity ranges from 20 ppt in winter to 32 ppt in summer. The surface area of the lagoon is 1.8 ha and the maximum depth is 3 m. Marine sticklebacks in Oyster Lagoon face a wide variety of predators [22]. Birds, such as great blue herons, mergansers and kingfishers, are the primary piscivores in the lagoon. Mammals (river otters, racoons, mink), and reptiles (e.g. Pacific garter snakes) are also potential predators of sticklebacks. Sculpins also feed on adult and juvenile sticklebacks in the lagoon [22]. The marine fish from Oyster Lagoon have a high-plated phenotype [30], which has been associated with high predation by vertebrate predators [31]. Freshwater adult fish were collected from Hoggan Lake on Gabriola Island (49°09′08′′N 123°49′′W). The lake is not connected to the ocean, and this population has been isolated from marine ancestors for approximately 10,000 years. It covers a surface area of 19.7 ha and has a perimeter of 2.2 km. The lake does not contain sculpin, a known predator of sticklebacks, but fish stocking records indicate that it was stocked with rainbow trout (*Oncorhynchus mykiss*) and coastal cutthroat trout (*Oncorhynchus clarkii clarkii*) in 1927 [32] with both of these species being intermittently observed in this environment, indicating that either stocked fish established a self-sustaining population, or that the lake has always contained a natural population. There is no known benthic-limnetic phenotypic divergence in this population. The fish from this population have a low number of lateral armour plates [13], which has been associated with low predation regimes [31].

Adult threespine sticklebacks were crossed to produce pure lines of F1 individuals, resulting in 14 full-sub F1 families, seven from each population. Juveniles were kept in 110 L-tanks under a 12L:12D cycle, with temperature at 18°C in the Life and Science Animal Research Centre at the University of Calgary and fed frozen bloodworms to satiation twice daily. Salinity was maintained at 5 ppt to promote health and maintain proper development in both marine and freshwater stickleback (see [33] for a similar rearing protocol of marine and freshwater populations). Juveniles (8-month old) were air-shipped to the “Laboratoire de Recherche en Sciences Aquatiques” at Université Laval. Juveniles were held in separate 80 L-tanks (stocking density: 0.11 g/L) under similar rearing conditions with the exception that all fish were acclimated to 15°C with a 8L:16D photoperiod (winter conditions). Juveniles from all families originating from a given environment (marine or freshwater) were kept together during air transport and later rearing, such that the information about the family of origin of individuals is not available. All fish were in a non-reproductive state (body length: average of 46.4±0.6 mm for fish from Hoggan Lake; 42.5±0.5 mm for fish from Oyster Lagoon) and sex was determined using a genetic sex marker [34].

### Behavioural series

**Figure 1:**
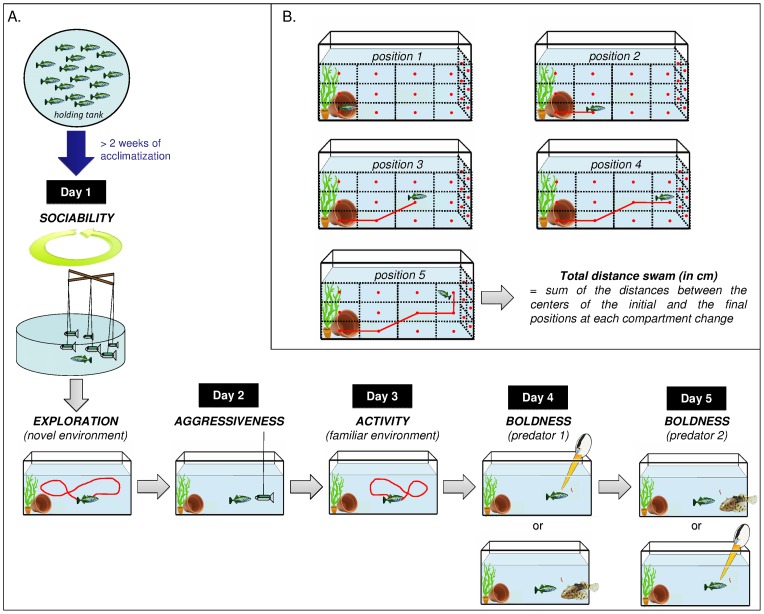
Illustrations of (A) the set-ups and the timeline of the experiment, and (B) the method to calculate the total distance swam of each fish in the exploration and the activity trials.

Behavioural experiments began after at least two weeks of acclimatisation. Each individual fish (n = 36 fish/population) was exposed to five behavioural tests over a 5-day period (sociability, exploration, aggressiveness, activity and boldness, see Fig. 1A for a timeline). After the sociability assay, the focal individual was transferred to an individual 45 L-experimental tank with plants and shelter as enrichment. The focal individual stayed in this tank for the rest of the experiment. In total, there were 6 series of 5 days (6 fish per population tested per series; 12 fish per series). All tests were done at the same time of day for all fish. Repeatability of these behaviours has already been demonstrated in the threespine stickleback [35], [36]. Each behavioural assay was filmed with a digital camera (JVC model GZ-MS120) mounted in front of the tank. The tanks were drained, cleaned and filled with water again between each series.

#### Sociability

We used an assay previously described in [21] that ensures repeatable stimulus conditions and the absence of confounding effects of the behaviour of the conspecifics in the school. A motorised mobile reproducing a school of five fish with the same colour as juvenile sticklebacks and of a smaller body length than the average size of the populations (36 mm) was constructed. The artificial school was immersed 10 cm from the water surface and from the tank bottom in an 80 L-tank. The focal fish was added in the tank in the presence of the motionless school for a 15-minute acclimatisation period. The rotating motor connected to the mobile then moved the fish school around the tank for 15 minutes. The focal fish's schooling behaviour was filmed from above. The time spent swimming within one body length of the group in the same direction and at the same speed (in seconds) was quantified as sociability.

#### Exploration

Immediately after the sociability test, the focal fish was moved into its novel individual experimental tank, where it remained throughout the rest of the behavioural experiment. The fish was placed in a 7 cm-diameter opaque cylinder with an open end, which was then introduced into the tank. The exploratory behaviour in this novel environment was quantified using three measures: (1) latency to exit the cylinder (in seconds) was measured (if the fish was still inside the cylinder after ten minutes following introduction, the cylinder was removed and it was given the maximum possible latency score, i.e. 600 sec), (2) after cylinder removal, time spent swimming (in seconds) in the new environment, and (3) total length swam (in cm) were measured for 5 minutes. The swimming activity of each fish was observed on the video recording and the total distance swam quantified manually using a 3-D grid divided into 4×2×3 compartments. Each time the fish swam from a compartment to another, the distance between the centers of the initial and the final compartments was measured (Fig. 1B). All distances were summed-up over the 5-min period.

#### Aggressiveness

On the second day, a model juvenile stickleback fish (body length: 36 mm, colour: olive green and grey) was placed as an intruder in the observation tank and the resulting interactions were quantified for a period of 15 minutes. The number of bites made by the focal fish against the intruder was used as a measure of aggressiveness [37]. The percentage of individuals exhibiting at least one biting event was also quantified.

#### Activity

Activity is a measure of the general level of swimming activity in its familiar environment, in contrast to exploration, which is measured in a novel environment. On the third day when the fish was familiarised with its experimental tank, the time spent moving (in seconds), as well as the total length swam (in cm) were measured, as previously explained for the exploration assay, for each subject during a 5-minute period, at the same time of day as in the exploration test.

#### Boldness

The response of an individual in a risky situation was used as a measure of boldness. On the fourth and fifth day, during the feeding session, a simulated predator attack was manually performed by the experimenter on the focal fish using a replica of the head of a great blue heron or a flexible lure in the shape of a sculpin fish, two natural predators of sticklebacks. The feeding session started with provision of a single bloodworm, followed by a 30-second wait and a second bloodworm. The predator attack was simulated at the third bloodworm, when the focal fish approached within at least one body length of the food. Striking the water with the beak directly over the area where food was distributed simulated the sudden overhead attack of a heron searching for prey. The beak was plunged about 5 cm below the water surface and moved in the water for 10 seconds. To simulate a large sculpin attack, the sculpin replica was suddenly placed outside the front of the aquarium and moved along the length of the tank for 10 seconds. The experimenter was hidden behind a black curtain, such that no human movement was perceptible to the fish. Latency to resume feeding after the attack (in seconds) was used as a measure of boldness. Each focal fish was tested for each predator on two days (one predator per day). The order of appearance of the two predators was randomised for each fish to minimise carry over effects.

### Data availability

The entire dataset used is available as Table S1.

### Data analysis

All statistical analyses were performed using R software 3.0.2 (http://cran.r-project.org/bin/windows/base/). Normal distribution of data and homogeneity of variances were assessed using Shapiro-Wilk and Bartlett tests respectively; however, the data did not meet the assumption of normality. Because the data was not normally distributed, we used a linear model for each behaviour, with p-values obtained by permutation tests (lmPerm package, Permutation tests for linear models in R). These models were used to test for behavioural differences between the two populations including population, sex and length of the fish as fixed effects. For boldness, the significance of interactions between the effects of population, predator type, day of tests, as well as fish identification number (ID) were tested in order to correct for pseudo-replication (as individuals were tested twice on the fourth and fifth days). Only population effects are reported in the text as no effect of sex, fish length, day and fish ID was found on any behaviour tested. Median ± quartiles are presented and p<0.05 was used as the statistical significance threshold (see Table S1 for the dataset).

For each population separately, Spearman's rank correlations were calculated between body length of fish, and each of the eight behavioural parameters measured. No correlation was found with body length and these terms were subsequently removed from the analysis. Correlations between behaviours were also quantified using Spearman's rank correlations. As multiple correlations were performed, sequential Benjamini and Hochberg corrections [38] were applied to reduce type I errors by controlling for the false discovery rate. Correlations were found between different behaviour components within a population. However, after correcting for multiple testing, the only correlations that were significant were found between different measures of the same behaviour (see Table S2).

## Results

### Average behavioural divergence between populations

#### Sociability

**Figure 2:**
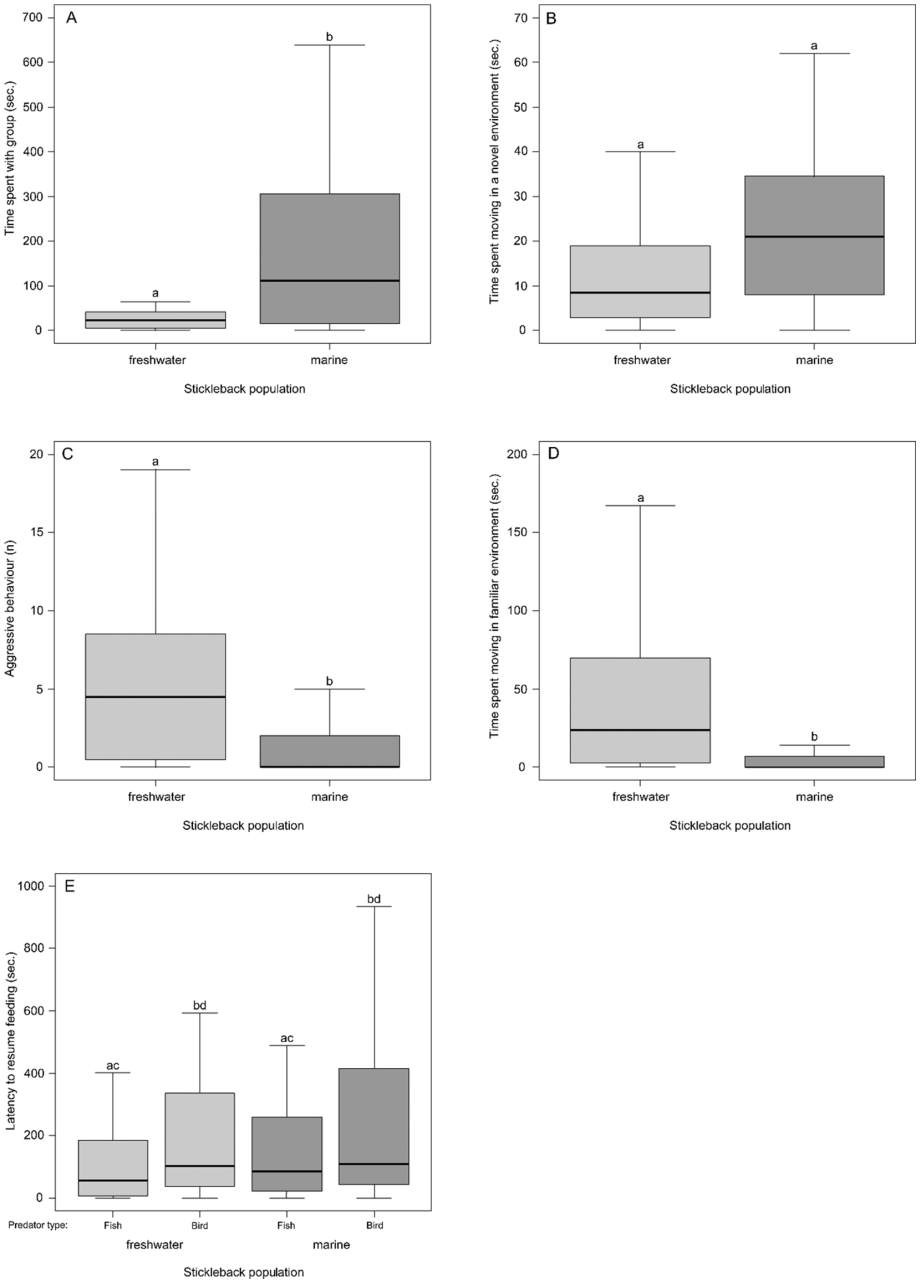
Behaviour (median ± quartiles) of juvenile threespine sticklebacks from a marine and a freshwater population. (A) Sociability, (B) exploratory behaviour in a novel environment, (C) aggressiveness, (D) activity in a familiar environment, and (E) boldness. Different letters indicate a significant difference between populations.

The two populations exhibited different schooling tendencies under identical social circumstances (Table 1 & Fig. 2A). The model school assay elicited schooling behaviour for 85% of individuals in the freshwater population and for 90% of the individuals in the marine fish. During the entire 15 min-session, freshwater fish spent an average of 4.4% of their time schooling (mean  = 39.4 sec; SEM: ±9.9) while marine fish spent 21% of their time grouping with the school (mean  = 188.9 sec; SEM: ±35.5; Fig. 2A). Accordingly, freshwater sticklebacks showed a significantly lower schooling behaviour than the marine fish (Linear model: population term, p = 0.002, n = 35 marine fish, n = 35 freshwater fish).

**Table 1:** Summary of linear model statistical tests conducted to compare the two juvenile threespine stickleback populations for five behaviours.

Behaviours	Variable	Estimate	P-value	Population trends
Sociability	population	−70.782	0.011*	marine > freshwater
	sex	3.410	0.922	
	length	−1.912	0.784	
Exploration – latency to exit cylinder	population	13.159	0.902	marine = freshwater
	sex	10.506	0.638	
	length	6.331	0.302	
Exploration – time spent swimming	population	−3.626	0.745	marine = freshwater
	sex	3.285	0.510	
	length	−0.878	0.317	
Exploration – total length swam	population	0.332	0.961	marine = freshwater
	sex	−1.132	0.961	
	length	1.921	0.312	
Aggressiveness	population	2.297	0.002**	marine < freshwater
	sex	−0.970	0.270	
	length	−0.220	0.265	
Activity – time spent swimming	population	19.573	0.002**	marine < freshwater
	sex	−3.572	0.388	
	length	−0.522	0.638	
Activity – total length swam	population	40.187	0.013*	marine < freshwater
	sex	−18.725	0.171	
	length	−0.002	1.000	
Boldness	population	−46.004	0.961	marine = freshwater
	sex	−0.838	1.00	
	length	1.965	1.00	
	day	−31.193	0.863	
	fish_ID	−0.419	0.863	
	predator type	−53.608	0.041*	

Estimate and p-value probability obtained using the lmp function of the lmPerm package in R. Asterisk (*) denotes significant difference.

#### Exploration

Exploration in a novel environment was not significantly different between populations for the three measures observed (Linear model: population term, p>0.05 for latency to exit a refuge, n = 32 marine fish, n = 34 freshwater fish; total distance swam, n = 33 marine fish, n = 31 freshwater fish; and time spent moving, n = 35 marine fish, n = 32 freshwater fish; Table 1 & Fig. 2B).

#### Aggressiveness

About 75% and 39% of the fish exhibited aggressiveness in the freshwater and marine population respectively, whereas the rest did not show a reaction to the intruder (i.e. no approach within one body length). When comparing all individuals tested, the number of bites was four times higher in the freshwater than in the marine population (Linear model: population term, p = 0.002, n = 26 marine fish, n = 32 freshwater fish; Table 1 & Fig. 2C).

#### Activity

Activity in a familiar environment measured as time spent moving was five times higher for freshwater individuals than marine ones (Linear model: population term, p = 0.0006 for time spent moving, n = 36 marine fish, n = 36 freshwater fish; Table 1 & Fig. 2D) and a significant difference was also found for total distance swam (p = 0.010, n = 36 marine fish, n = 36 freshwater fish; Table 1).

#### Boldness

In the boldness under predation risk test, 94% of freshwater fish responded to the heron predator attacks and 79% to the sculpin by hiding in the shelter, while 97% of marine fish showed this reaction for both predators. The two populations did not differ in boldness; all fish exhibited the same average latency to resume feeding after a predator stimulus (Linear model: population term, p = 0.961, n = 32 marine fish, n = 35 freshwater fish for the fish predator, n = 33 marine fish, n = 35 freshwater fish for the bird predator; Table 1 & Fig. 2E). Fish responded differently to the type of predator in both populations (Linear model: predator type term, p = 0.041; no significant population X predatory type interaction). The bird-shaped predator induced the longest latency in both populations (Fig. 2E).

## Discussion

We found that common-environment reared and predator-naïve juvenile threespine sticklebacks originating from a freshwater habitat were less social, more active in a familiar environment and more aggressive towards conspecifics than their marine conspecifics. Given that all adult fish were bred in the laboratory and that juveniles were raised in the same setting without parental care, our results suggest that a genetic difference may underlie the measured divergence in behaviour; although further investigations are needed to confirm this hypothesis. It has been shown that boldness [28], [39], aggressiveness [27], [28], [39], exploration in an unfamiliar environment [28], [39] and activity in a familiar environment [27], [28], [39] among others show genetic variation between freshwater populations in threespine stickleback, while schooling behaviour has been shown to differ between marine and freshwater populations of this species [21]. We thus uncover important behavioural differences between individuals belonging to the ancestral marine population and the freshwater one for two previously under- or unstudied ecologically-relevant behaviours, i.e. activity and aggressiveness, in addition to confirming divergence in sociability, in line with what has been found previously in another population pair [21].

Our results suggest that the behavioural divergence observed could represent the result of natural selection that acted on individuals after freshwater colonisation [40]. However, the hypothesis that this phenotypic divergence could result from other causes than selection cannot be excluded. Neutral processes acting during and after colonisation on genetic diversity of the population, e.g. genetic drift, could explain the divergence [41], although the findings that sociability diverges in the same direction as what was found previously for other stickleback populations [21] and other species [11] tends to support the hypothesis of the effects of natural selection. The evidence gathered demonstrating the parallel evolution of various traits (morphology, physiology) occurring in hundreds of geographically and genetically isolated freshwater populations of threespine stickleback after colonisation from the marine environment speaks in favour of natural selection [42]. Our study provides additional evidence supporting the hypothesis of repeated phenotypic divergence when facing similar ecological challenges, as we find the same significantly lower sociability tendency in freshwater fish compared to marine fish as previously found for entirely different marine (Pacific Ocean in Japan) and freshwater populations (benthic population from Paxton Lake in Canada) of this species [21]. This repeated strong behavioural difference associated with colonisation of the freshwater environment supports the hypothesis that this divergence in behaviour enhances fitness in the new ecological conditions. Of course, other stickleback marine-freshwater population pairs must be characterised for their behavioural phenotypes in both habitat types in order to confirm that behavioural differences measured in this study between a marine and freshwater population were due to divergent natural selection rather than being merely difference between any two populations, regardless of ecological pressures.

Predation pressure is one of the main ecological factors that act as agents of selection, which can cause phenotypic divergence among prey populations [1]. In threespine stickleback, contrasting predation pressures between marine and freshwater populations are correlated with divergence in lateral plate armour development [31]. The marine and freshwater habitats from which breeding adults were sampled to create the two juvenile laboratory lines studied differed in predation regime (based on armour morphology, see methods) thus suggesting that predation could be a selection agent acting differentially on behaviour in the marine and the freshwater population, similar to what is found for predator defence morphology [31]. Indeed, studies comparing freshwater populations showed an association between predatory regime differences and divergence in behaviour in sticklebacks in sociability [25], activity [26], [27], aggressiveness [27], [28], and boldness [26], [28], [29]. The strength of our results, although based on only two populations, is that they are in accordance with the predictions of behavioural divergence in sociability, aggressiveness, and activity between marine and freshwater fish we made *a priori* based on these studies of freshwater populations. For example, in a high-predation habitat such as the marine environment, schooling provides fish with several benefits such as a better foraging efficiency and a greater antipredator defence [43]. It has been hypothesised to lead to a reduction of aggression between individuals because high levels of fighting might offset the benefits of schooling [44]. However, we keep in mind that marine and freshwater habitats differ in several other ecological aspects, such as habitat structure and stability [26], [45], food resource availability [46], intraspecific competition [47] and temperature [30], which could also explain the behavioural divergence observed. The fact that boldness did not differ between populations contrary to our prediction, and that both populations reacted more to the bird predator, suggests that other factors must also be considered. The behaviour of freshwater juveniles could also represent heritable behavioural characteristics of the marine individuals that preferentially invaded freshwater after the glacial retreat to form the new lake population. A greater swimming activity and a higher boldness could be associated with a higher propensity to use novel habitats [48], [49]. The freshwater individuals were significantly more active than marine fish, although they did not differ in boldness. This is in line with the hypothesis that the freshwater behaviour stems from standing genetic variation in the marine population, as very bold individuals could also be found in the marine population, as observed in previous studies for morphological traits in threespine stickleback [50]. In this case, natural selection acting on these individuals once in the new freshwater environment is also possible.

Our study suggests that mean behaviours in a population are associated with their own costs and benefits modulated according to the habitat. Taken together, our results provide an integrated view of the divergence in mean behaviour of individuals colonising a novel environment, potentially resulting from the new ecological constraints encountered, in contrast with the behaviour of the ancestral population, information which are of crucial importance considering the role of behavioural phenotype-environment associations in adaptive divergence.

## Supporting Information

Table S1 **Dataset used for the study by Di Poi et al.**
(TXT)

Table S2 **Statistically significant correlations found between behaviours in each population.** Correlation coefficients and p-values calculated before and after corrections for multiple testing (Benjamini-Horchberg) are presented.
(TXT)
